# MSH2/BRCA1 expression as a DNA-repair signature predicting survival in early–stage lung cancer patients from the IFCT-0002 Phase 3 Trial

**DOI:** 10.18632/oncotarget.14025

**Published:** 2016-12-19

**Authors:** Guénaëlle Levallet, Fatéméh Dubois, Pierre Fouret, Martine Antoine, Solenn Brosseau, Emmanuel Bergot, Michèle Beau-Faller, Valérie Gounant, Elisabeth Brambilla, Didier Debieuvre, Olivier Molinier, Françoise Galateau-Sallé, Julien Mazieres, Elisabeth Quoix, Jean-Louis Pujol, Denis Moro-Sibilot, Alexandra Langlais, Franck Morin, Virginie Westeel, Gérard Zalcman

**Affiliations:** ^1^ Service d’Anatomie et Cytologie Pathologique, Centre Hospitalier Universitaire de Caen, Normandie Université, Caen, France; ^2^ Normandie Université; UMR 1086 INSERM, Caen, France; ^3^ Service d’Anatomie Pathologique, Pitié-Salpêtrière, AP-HP, Université Pierre et Marie Curie (UPMC), Paris, France; ^4^ Service d’Anatomie Pathologique, Hôpital Tenon, AP-HP, Université Pierre et Marie Curie (UPMC), Paris, France; ^5^ Service de Pneumologie et Oncologie Thoracique, Centre Hospitalier Universitaire de Caen, Normandie Université, Caen, cedex, France; ^6^ Service d’Oncologie Thoracique, Hôpital Bichat-Claude Bernard, AP-HP, Université Paris-Diderot, Paris, France; ^7^ Laboratoire de Biochimie et Biologie moléculaire, Hôpital de Hautepierre, Centre Hospitalier Universitaire de Strasbourg, Université de Strasbourg, BP426 Strasbourg, Cedex, France; ^8^ Service de Pneumologie, Hôpital Tenon, AP-HP, Université Pierre et Marie Curie (UPMC), Paris, France; ^9^ Service d’Anatomie Pathologique, Hôpital Albert Michallon, Centre Hospitalier Universitaire de Grenoble, La Tronche, France; ^10^ Service de Pneumologie, GHRMSA-Centre Hospitalier Eric Müller, Mulhouse, France; ^11^ Service de Pneumologie, Centre Hospitalier du Mans, Le Mans, France; ^12^ Service de Pneumologie, Hôpital Larrey, Centre Hospitalier Universitaire de Toulouse, Université de Toulouse III, Toulouse, France; ^13^ Service de Pneumologie, Nouvel Hôpital Civil, Hospices Civils de Strasbourg, Université de Strasbourg, BP426 Strasbourg, Cedex, France; ^14^ Service de Pneumologie, Centre Hospitalier Universitaire Arnaud de Villeneuve, Université de Montpellier, Montpellier, France; ^15^ Unité d’Oncologie Thoracique–Pneumologie, CHU de Grenoble, INSERM U823, Grenoble, France; ^16^ Intergroupe Francophone de Cancérologie Thoracique (IFCT), Paris, France; ^17^ Service de Pneumologie, Hôpital Jean-Minjoz, Centre Hospitalier Universitaire de Besançon, Université de Franche-Comté, Besançon, France; ^18^ CIC INSERM 1425-CLIP2 Paris-Nord, Hôpital Bichat-Claude Bernard, AP-HP, Paris, France; ^19^ On Behalf of the IFCT (Intergroupe Francophone de Cancérologie Thoracique)

**Keywords:** non-small cell lung cancer, neo-adjuvant chemotherapy, MSH2, BRCA1, MGMT

## Abstract

**Introduction:**

DNA repair is a double-edged sword in lung carcinogenesis. When defective, it promotes genetic instability and accumulated genetic alterations. Conversely these defects could sensitize cancer cells to therapeutic agents inducing DNA breaks.

**Methods:**

We used immunohistochemistry (IHC) to assess MSH2, XRCC5, and BRCA1 expression in 443 post-chemotherapy specimens from patients randomized in a Phase 3 trial, comparing two neoadjuvant regimens in 528 Stage I-II non-small cell lung cancer (NSCLC) patients (IFCT-0002). O6MGMT promoter gene methylation was analyzed in a subset of 208 patients of the same trial with available snap-frozen specimens.

**Results:**

Median follow-up was from 90 months onwards. Only high BRCA1 (n = 221, hazard ratio [HR] = 1.58, 95% confidence interval [CI] [1.07-2.34], p = 0.02) and low MSH2 expression (n = 356, HR = 1.52, 95% CI [1.11-2.08], *p* = 0.008) significantly predicted better overall survival (OS) in univariate and multivariate analysis. A bootstrap re-sampling strategy distinguished three patient groups at high (*n* = 55, low BRCA1 and high MSH2, median OS >96 months, HR = 2.5, 95% CI [1.45-4.33], *p* = 0.001), intermediate (n = 82, median OS = 73.4 *p* = 0.0596), and low (high BRCA1 and low MSH2, *n* = 67, median OS = ND, HR = 0.51, 95% CI [0.31-0.83], *p* = 0.006) risk of death.

**Interpretation:**

DNA repair protein expression assessment identified three different groups of risk of death in early-stage lung cancer patients, according to their tumor MSH2 and BRCA1 expression levels. These results deserve prospective evaluation of MSH2/BRCA1 theranostic value in lung cancer patients treated with combinations of DNA-damaging chemotherapy and drugs targeting DNA repair, such as Poly(ADP-ribose) polymerase (PARP) inhibitors.

## INTRODUCTION

Non-small cell lung cancers (NSCLC) account for approximately 85% of lung cancers. Despite curative-intent surgical resection, 20% of Stage I NSCLC patients die within 5 years, even following adjuvant chemotherapy, as NSCLC is highly metastatic and frequently chemo-resistant [1]. However, complete tumor resection followed by adjuvant platinum-based chemotherapy plays a central role as a curative treatment for NSCLC. The benefit offered by adjuvant chemotherapy is modest, with an absolute improvement in 5-year overall survival (OS) ranging from 4 to 15% [2–4]. The French Cooperative Thoracic Intergroup (IFCT) initiated a large Phase 3 trial in 2001 to evaluate *i)* tolerance and efficacy of gemcitabine-cisplatin versus paclitaxel-carboplatin perioperative chemotherapies; *ii)* the potential prognostic molecular biomarkers that could be helpful in defining therapeutic options and identifying genes/pathways that could be therapeutically targeted [5]. Lethal cisplatin-induced cell injury was extensively studied *in vitro*: cisplatin binds to DNA and induces adducts by covalent cross-linking between DNA strands, thereby inhibiting DNA replication and leading to cell apoptosis. Nevertheless, DNA repair mechanisms that reduce the effectiveness of cisplatin can ensure tumor-cell survival. The way to overcome this resistance is to combine cisplatin with a molecule inducing cell toxicity through alternative mechanisms, such as gemcitabine (2′,2′-difluoro-2′-deoxycytidine), a nucleoside analogue, which interferes with DNA synthesis, or paclitaxel, a tubulin depolymerization-inhibitor, which alters the mitotic spindle by stabilizing microtubules. Combination platinum-based chemotherapy achieves 30-40% 1-year survival rates in Stage IV NSCLC and has proven superior to single agents or best supportive care in this setting. Gemcitabine inhibits the repair of cisplatin-induced intrastrand adducts and interstrand cross-links [6]. Paclitaxel, by preventing microtubule depolymerisation, blocks the cell cycle progression in late G2-M phases. It has also been proposed that taxanes (docetaxel and paclitaxel) can induce DNA single-strand breaks (SSB), depending on the cell type [7,8]. The DNA damage repair process has crucial implications and, depending on their DNA repair efficiency, cancer cells can: *i)* interrupt the cell cycle to repair the DNA damage*, ii)* commence apoptosis, or *iii)* proceed with mitosis and cell proliferation without repairing the damage (while more molecular alterations accumulate). While the TP53 gene product has been shown as a key guardian of genome integrity [9], specific enzymes involved in genome integrity survey or DNA damage repair have been described, including key DNA-repair proteins such as XRCC5, MSH2, BRCA1, and O^6^MGMT, respectively involved in nucleotide excision repair (NER), base excision repair (BER), mismatch repair (MMR), or non-homologous end-joining (NHEJ) systems. These enzymes have been previously studied individually in NSCLC patients to assess their prognostic or predictive roles [10–23]. In response to the lack of consensus in the literature regarding the value of these enzymes’ expression in tumors as predictive biomarkers in NSCLC [24], the IFCT 0002 Phase 3 randomized trial, with its large patient sample (528 patients enrolled between 2001 and 2005) and the homogeneity of their treatments, constituted further opportunity to assess whether or not XRCC5, MSH2, BRCA1, and O^6^MGMT represent reliable biomarkers in Stage I and II NSCLC patients, treated with taxane- or anti-metabolite-based perioperative chemotherapy.

## RESULTS

### DNA repair protein alterations and patient characteristics

**Figure 1:**
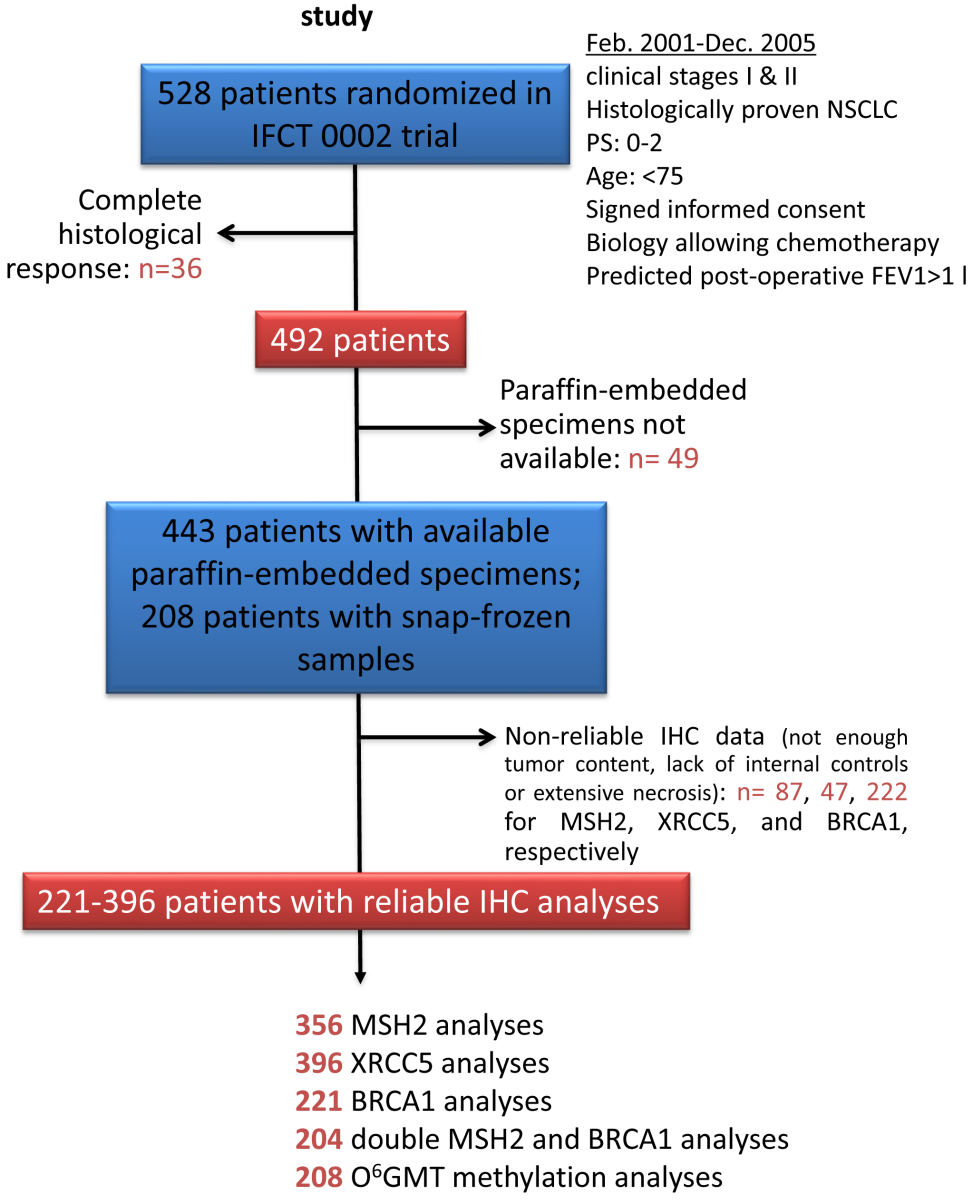
Patients and histological sample disposition in the Bio-IFCT 0002 study

**Figure 2:**
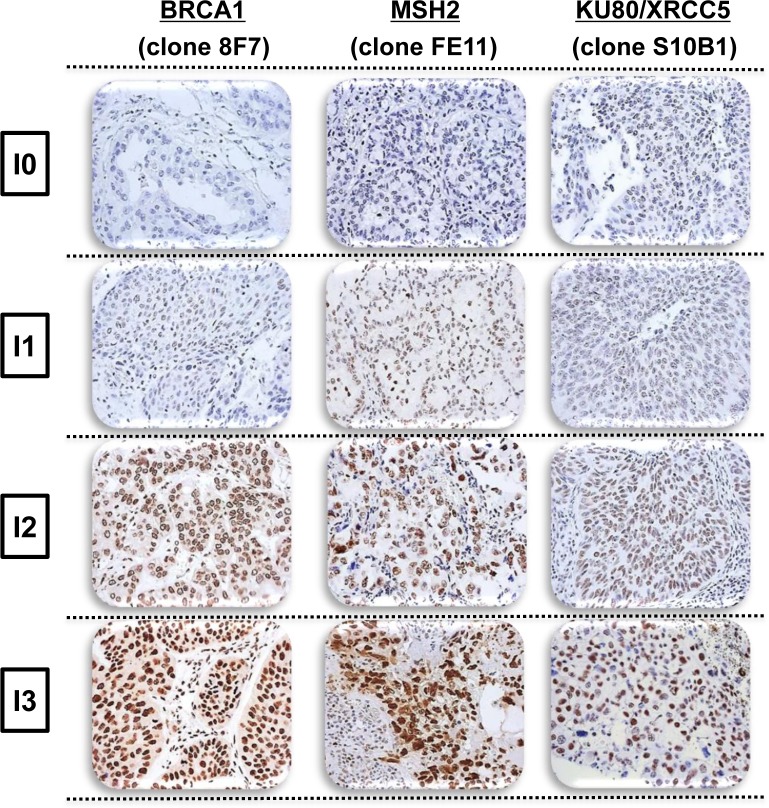
Representative intensity of BRCA1, MSH2, and XRCC5 immunostaining in non-small cell lung cancer, demonstrating negative (I = 0), weak (I = 1), moderate (I = 2), or strong (I = 3) staining

MSH2, XRCC5, and BRCA1 tumor immunostaining assays were technically possible for 356 (77.2%), 396 (85.9%), and 221 (47.9%) patients with no complete histological response, respectively (Figure 1), revealing specific nuclear staining on a slide containing substantial tumor content, without extensive necrosis (Figure 2). Staining intensity varied markedly between lung-cancer samples and within the same slide, with strongly-stained clusters of tumor cells sometimes observed adjacent to weakly-stained tumor cells.

The characteristics of the IFCT-002 subset patients with IHC analyses have previously been described [25], presenting a mean age of 60.0 years (SD: 9.1, range: 35-76 years) and Eastern Cooperative Oncology Group (ECOG) performance status (PS) of 0 (77.2-77.8%). Only 9.7 to 10.2% of patients were “light” smokers (<10 packs per year), and 51.1 to 56.3% had non-squamous histology. The 396 patients with at least one DNA repair protein analysis available exhibited higher probability of having non-squamous NSCLC (*p* <0.0001), though no significant difference was observed with the 132 patients without DNA-repair protein IHC analyses for other characteristics. They especially exhibited similar OS and DFS values [25]. Of the 208 snap-frozen specimens, O^6^MGMT promoter methylation was found in 14.9%, and this subset was also characterized by a higher frequency of non-squamous histology (46.2% *vs*. 32.8%, *p* = 0.0051) in comparison with the rest of the population [26].

### O^6^MGMT methylation and XRCC5 expression do not influence survival

The average XRCC5 expression intensity score was 25.07 ± 25.35, with a median of 20 [10–30]. Neither O^6^MGMT methylation, nor XRCC5 expression either dichotomized at the median value or studied as a continuous variable, had any impact on OS and DFS of early-stage NSCLC patients in Cox models (data not shown).

### High MSH2 expression significantly predicts worse overall survival

The average MSH2 expression intensity score was 231.03 ± 69.09, with a median of 255 [180–300]. MSH2 staining was first studied as a continuous variable. Low MSH2 staining was more frequent in young (*p* = 0.03) and ECOG PS 0 patients (*p* = 0.008) (Table 1). MSH2 status did not significantly differ according to gender, treatment arm, number of chemotherapy cycles, histological differentiation, pathological stage, smoking status, or clinical T. MSH2 staining, analyzed as a continuous variable, and tested for a 1 unit increase of IHC score (from 0 to 300), significantly predicted OS in univariate analysis (HR = 1.003, 95% CI [1.000-1.005]; *p* = 0.020) and multivariate backward model (adjusted HR = 1.003, 95% CI [1.000-1.005]; *p* = 0.03), including all variables with a p-value <0.2 in the univariate analysis (gender, Stage I *vs*. II, and squamous *vs*. non-squamous histology) (Table 2).

**Figure 3:**
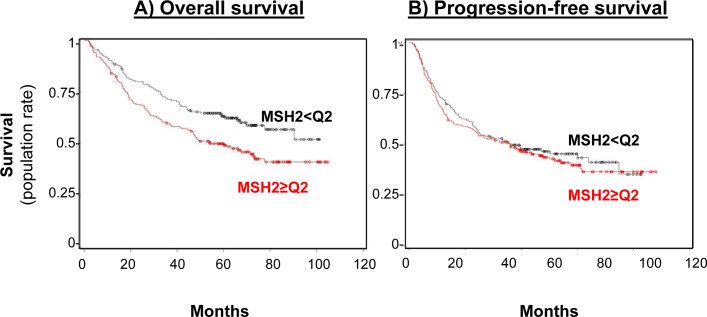
MSH2 level impact on both overall survival (A) and progression-free (B) survival rates **A.** Overall survival curve for the 356 patients in the bio-IFCT 0002 trial, according to MSH2 expression. **B.** Progression-free survival rate curve for the 356 patients in the bio-IFCT 0002 trial, according to MSH2 expression.

**Figure 4:**
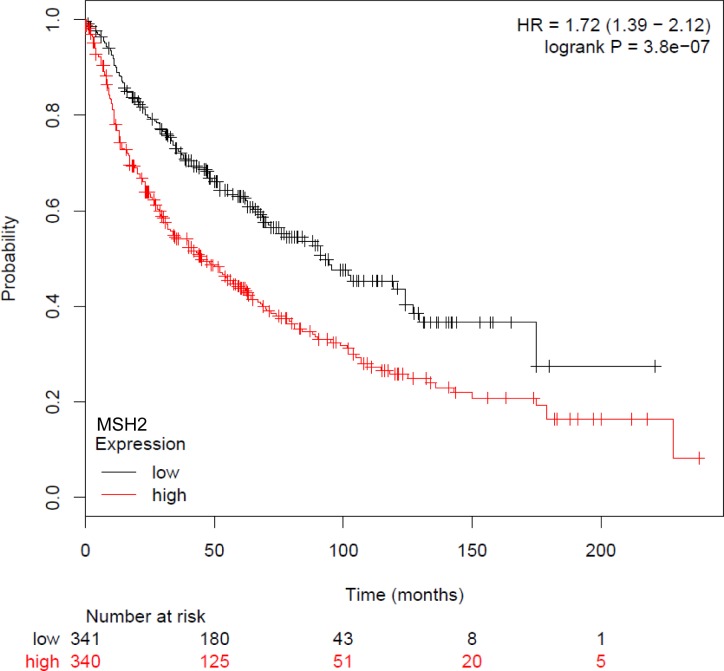
Kaplan-Meier overall survival curves according to MSH2 mRNA, from GEO (Affymetrix microarrays only), EGA, and TCGA databases (2015 database release), *using kpm.plot.com* online software, in 681 NSCLC Stage I-III patients

Subsequent statistical analyses, correlating MSH2 expression with DFS or OS, have dichotomized the MSH2 score, with low (score below the median of 255, *n* = 167 [46.9%]) and high MSH2 expression (score above the median, *n* = 189 [53.1%]). Patients whose tumor samples expressed low MSH2 had not reached the median OS at the time of follow-up, compared to patients whose tumor samples expressed high MSH2 (60.5 months [44.6–77.8]) (Figure 3, left panel), in both univariate (HR = 0.65 [0.48 to 0.89], log-rank *p* = 0.007) and multivariate analysis (Cox model, including stage, histology, and gender, adjusted HR = 0.66, 95% CI [0.48-0.90], *p* = 0.008) (Table 2). DFS was not significantly affected according to MSH2 level (Table 2 and Figure 3, right panel). Finally, MSH2 influence on prognosis did not differ according to treatment arm (paclitaxel versus gemcitabine), since no significant interaction was observed between MSH2 expression status and treatment arm for DFS prediction (data not shown). For external validation of our data, we used kpm.plot.com online software, computing the MSH2 mRNA prognostic analyses in 681 Stage I-to-III patients, with gene-expression data and OS information downloaded from the GEO (Affymetrix microarrays only), EGA, and TCGA databases (2015 database release) [27]. OS analysis was dichotomized according to the median value (Figure 4) and revealed high MSH2 mRNA content to also be significantly associated with poorer survival in this series of patients (HR = 1.72; 95% CI [1.39-2.12], *p* = 3.8×10^−7^)

**Table 1:** MSH2/BRCA1 Immunostaining Characteristics

Characteristics	MSH2 < Q2 n = 167	MSH2 ≥ Q2 n = 189	p	BRCA1 < Q2 n = 92	BRCA1 ≥ Q2 n = 129	p	MSH2 < Q2 + BRCA1 ≥ Q2 n = 67	Other n = 137	p
Gender									
Male	129 (77.2%)	152 (80.4%)	0.4631	72 (78.3%)	98 (76%)	*0.6901*	48 (71.6%)	106 (77.4%)	*0.3715*
Female	38 (22.8%)	37 (19.6%)		20 (21.7%)	31 (24%)		19 (28.4%)	31 (22.6%)	
Age at inclusion ≤60 years old >60 years old	91 (54.5%) 76 (45.5%)	77 (40.7%) 112 (59.3%)	0.0095	42 (45.7%) 50 (54.3%)	68 (52.7%) 61 (47.3%)	*0.3007*	38 (56.7%) 29 (43.3%)	64 (46.7%) 73 (53.3%)	*0.1796*
Pack-year * ≤10 >10	19 (11.4%) 147 (88.6%)	17 (9.1%) 170 (90.1%)	*0.4656*	9 (9.9%) 82 (90.1%)	12 (9.5%) 115 (90.5%)	*0.9133*	9 (13.6%) 57 (86.4%)	11 (8.1%) 125 (91.9%)	*0.2156*
Performance status									
0	139 (83.2%)	136 (72%)	***0.0113***	67 (72.8%)	105 (81.4%)	*0.1306*	57 (85.1%)	99 (72.3%)	***0.0428***
1 or 2	28 (16.8%)	53 (28%)		25 (27.2%)	24 (18.6%)		10 (14.9%)	38 (27.7%)	
Histology									
SCC	58 (34.7%)	82 (43.4%)	*0.0952*	31 (33.7%)	50 (38.8%)	*0.4412*	23 (34.3%)	55 (40.1%)	*0.4220*
Non-SCC	109 (65.3%)	107 (56.6%)		61 (66.3%)	79 (61.2%)		44 (65.7%)	82 (59.9%)	
Arm (ITT)									
gemcitabine 4 cycles PRE	41 (24.5%)	47 (24.9%)	*0.1944*	17 (18.5%)	33 (25.6%)	*0.5934*	18 (26.9%)	28 (20.4%)	*0.746*
gemcitabine 2 cycles PERI	35 (21.0%)	51 (27.0%)		23 (25.0%)	33 (25.6%)		15 (22.4%)	37 (27.0%)	
paclitaxel 4 cycles PRE	38 (22.8%)	49 (25.9%)		25 (27.2%)	32 (24.8%)		17 (25.4%)	36 (26.3%)	
paclitaxel 2 cycles PERI	53 (31.7%)	42 (22.2%)		27 (29.3%)	31 (24.0%)		17 (25.4%)	36 (26.3%)	
Number of cycles received									
≤2	99 (59.3%)	106 (56.1%)	*0.5425*	53 (57.6%)	68 (52.7%)	*0.4711*	37 (55.2%)	75 (54.7%)	*0.9485*
>2	68 (40.7%)	83 (43.9%)		39 (42.4%)	61 (47.3%)		30 (44.8%)	62 (45.3%)	
Stage									
0,I	91 (54.5%)	106 (56.1%)	*0.7628*	41 (44.6%)	73 (56.6%)	*0.0779*	40 (59.7%)	64 (46.7%)	*0.0814*
II, III, IV	76 (45.5%)	83 (43.9%)		51 (55.4%)	56 (43.4%)		27 (40.3%)	73 (53.3%)	
cT									
T1	112 (67.1%)	116 (61.4%)	*0.2642*	60 (65.2%)	82 (63.6%)	*0.8006*	45 (67.2%)	83 (60.6%)	*0.3613*
T2 ou T3	55 (32.9%)	73 (38.6%)		32 (34.8%)	47 (36.4%)		22 (32.8%)	54 (39.4%)	

* information missing for 3 patients

**Table 2:** MSH2 Expression, Progression-Free Survival, and Overall Survival in Patients from the Bio-IFCT 0002 Trial

Outcome		MSH2 < Q2 n =167	MSH2 ≥ Q2 n =189	p	BRCA1 < Q2 n =92	BRCA1 ≥ Q2 n =129	p	MSH2 < Q2 + BRCA1 ≥ Q2 n =67	Other n = 137	p
PFS	Number of events	97	120		92	69		32	86	
	Median (month)	36.1 [24.1-61.1]	35.4 [23.2-46.4]		16.2 [13.3-36.0]	54.2 [39.8-ND]		ND	32.5 [16.0-49.1]	
	HR (95% CI)	0.88 [0.67-1.14]	1	0.33	1.44 [1.01-2.04]	1	0.04	0.62 [0.42-0.94]	1	0.02
	Adj. HR‡ (95% CI)	-	-	-	-	-	-	0.63 [0.42-0.95]	1	0.03
OS	Number of events	66	102		49	51		21	70	
	Median (month)	ND	60.5 [44.6–77.8]		47.1 [28.1-ND]	90.6 [73.5-ND]		ND	66.5 [42.3-ND]	
	HR (95% CI)	0.65 [0.48-0.89]	1	0.007	1.58 [1.07-2.34]	1	0.02	0.50 [0.31-0.82]	1	0.006
	Adj. HR^‡^ (95% CI)	0.66 [0.48-0.90]	1	0.008	1.58 [1.07- 2.34]	1	0.02	0.51 [0.31-0.83]	1	0.006

‡ HR was adjusted according to histology, stage, and gender for OS; HR was adjusted according to histology and stage for PFS

### MSH2 expression is not altered by chemotherapy

Given that patient tumor samples were collected following neoadjuvant chemotherapy, and since we were not able to compare such samples with the diagnostic biopsies obtained before surgery, we raised the question of chemotherapy’s influence on MSH2 expression. We used cell lines reproducing the most frequent molecular alterations in NSCLC: H1299 (p53 deletion, RASSF1A methylation), H1650 (EGF-R mutation, RASSF1A methylation, p53 mutation, CDKN2A deletion), H1975 (EGF-R mutation, p53 mutation, R273H, PI3K mutation, CDKN2A deletion), and A549 (RASSF1A methylation, RasSer12, CDKN2A deletion, Lkb1/STK11 mutation). We also used two isogenic non-tumorigenic, immortalized, bronchial cells lines with a low number of molecular alterations (CDK4 and hTERT lentiviral-mediated overexpression), HBEC-3 (p16/Rb block), and HBEC-3RasV12 (p16/Rb block, Ras mutation).

**Figure 5:**
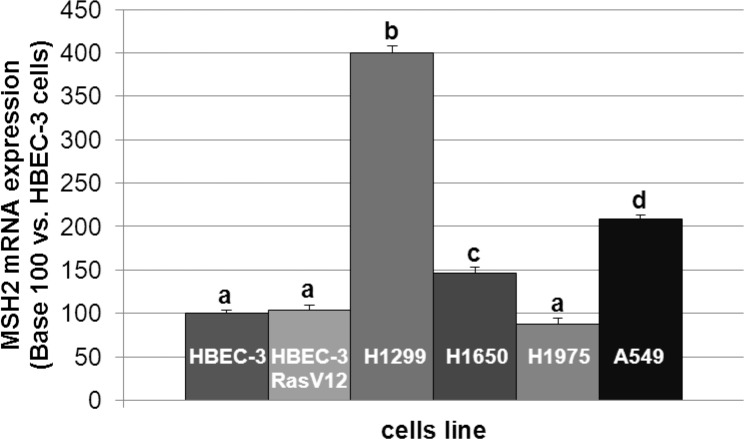
MSH2 mRNA expression level in lung cancer cell lines The different letters above the histograms represent the significant differences between them (one-way ANOVA, following post-hoc test of Fisher's LSD, *p* < 0.05).

**Figure 6:**
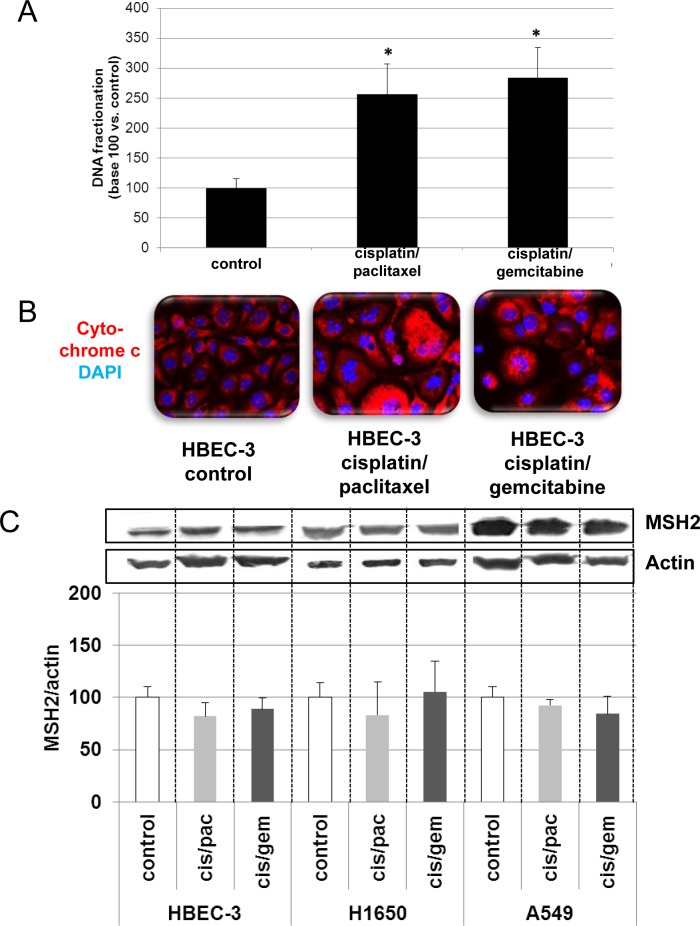
Effects of cisplatin/paclitaxel or cisplatin/gemcitabine treatment on DNA fragmentation (A), cytochrome C release (B), and MSH2 expression (C) in bronchial cell lines At 50% confluence, the bronchial cell lines were incubated or not (control) with cisplatin (2μM) for 3h before incubation for 48h with paclitaxel (10nM) or gemcitabine (250nM). Following treatment, DNA fragmentation, cytochrome C release, and MSH2 levels were measured. Results were expressed in base 100, with 100 attributed to the control cells (n = 3, t-test vs. control cells, *p <0.05).

**Figure 7:**
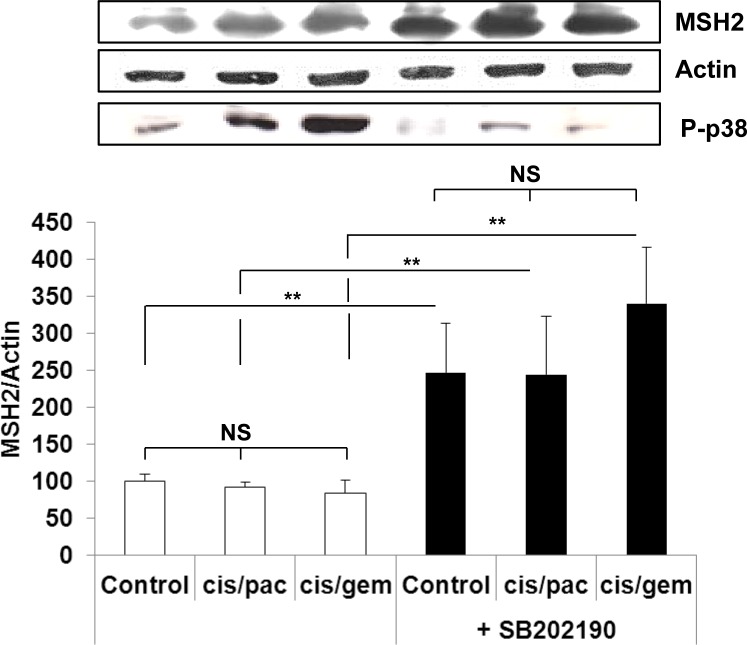
MSH2 expression following p38 blocking or cisplatin/paclitaxel or cisplatin/gemcitabine treatment in A549 cells At 50% confluence, bronchial cell lines were incubated or not (control) for 1h with SB202190 (20μM) then with cisplatin (2μM) for 3h before incubation for 48h with paclitaxel (10nM) or gemcitabine (250nM). Following treatment, MSH2 and phospho-p38 levels were measured by WB. Results were expressed in base 100, with 100 attributed to the control cells, *i.e.*, A549 without any treatment (one-way ANOVA, following post-hoc test of Fisher's LSD, **: *p* <0.01, NS: non-significant).

In line with the clinical trial findings (*i.e*., high MSH2 expression correlating with poor OS), we found that the cell lines expressing the highest MSH2 mRNA basal content were the H1299 and A549 cell lines (Figure 5), namely those with the highest tumorigenic potential when xenografted in nude mice [28–30]. Cells were incubated in the presence of cisplatin/gemcitabine or cisplatin/paclitaxel, as detailed in Materials and Methods. Treatment efficacy was evaluated by measuring DNA fragmentation and immunolabeling cytochrome C as readouts for cell apoptosis. In the HBEC-3 non-tumorigenic cell line, both cisplatin/paclitaxel and cisplatin/gemcitabine treatments significantly increased DNA fragmentation 2.5- to 2.8-fold (Figure 6A, t-test, treated cells *vs*. control cells, *p* <0.05), as in the HBEC-3-RasV12, A549, H1650, and H1299 cells (data not shown). Both treatments increased cytochrome c cytoplasmic signal nearly 2-fold in these cells (Figure 6B), again demonstrating chemotherapy-induced apoptosis in such conditions in non-tumorigenic and tumorigenic cell lines. Conversely, chemotherapy had no impact on MSH2 protein content, as demonstrated by the stable MSH2/actin ratios (Figure 6C). It has been suggested that such chemotherapy regimens could induce p38/Mitogen-Activated Protein Kina se14 (MAPK14) activity, a stress-activated signaling, which contributes to the cytotoxic effect of cisplatin [31–33]. Here, we reported that, despite the observed phospho-p38 increase (as a readout for p38 activation) induced by cisplatin (shown here for A549 cells in Figure 7), the MSH2 protein levels were not altered by chemotherapy (Figures 6C and 7) in HBEC-3 (non-tumorigenic), H1650 (EGFR mutation), and A549 (K-Ras mutation) cell lines, as with H1299 cell lines (data not shown). In the A549 cells, blocking p38 MAPK activation using pharmacological inhibitor SB202190 did not modify MSH2 expression, while A549 cells contained an inactivating STK11/LkB1 mutation, with Lkb1 inactivation leading to p38 hyper-activation [34]. These *in vitro* results suggest that neo-adjuvant chemotherapy did not alter MSH2 expression, and that MSH2 status was independent of p38 activity in tumors from patients of the Bio-IFCT0002 trial. In line with this data, we did not find that phospho-p38 intensity staining had any influence on OS and DFS of patients from the Bio-IFCT0002 trial, nor did treatment arm (data not shown).

### A new signature for high-risk recurrence and death

BRCA1 is another extensively studied DNA-repair protein. Allelic loss, mutations, and gene methylation have been shown to alter BRCA1 protein content. We also report that low BRCA1 expression significantly predicted both worse OS and PFS in early-stage NSCLC. The average BRCA1 expression intensity score was 103.98 ± 74.07, with a median of 100 [30-160]. As with MSH2, BRCA1 status was not significantly affected by the clinical parameters, either when low or high according to dichotomization at median value (Table 1).

**Figure 8:**
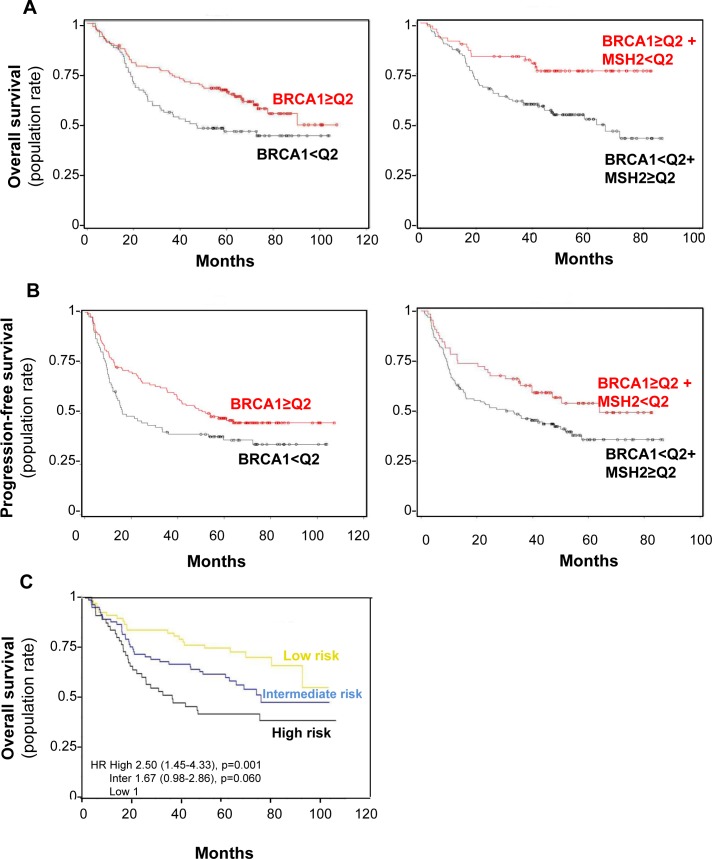
BRCA1 level impact on both overall survival (A) and progression-free (B) survival rates in combination with MSH2 level or alone **A.** Overall survival curve for the 221 patients in the bio-IFCT 0002 trial, according to BRCA1 with or without MSH2 expression. **B.** Progression-free survival rate curve for the 221 patients in the bio-IFCT 0002 trial, according to BRCA1 with or without MSH2 expression. **C.** Identification of three groups of early-lung cancer patients from low to high risk of recurrence, according to BRCA1 and MSH2 level. “High” risk of death: low BRCA1/ high MSH2 (*n* = 55); “low”: high BRCA1/low MSH2 (*n* = 67); “intermediate” for other combinations (*n* = 82).

To assess the influence of BRCA1 expression on DFS and OS, expression IHC scores were dichotomized at median value, with low score defined as below the median value of 100 (92 [41.6%] patients) and high score as above (129 [58.4%] patients). BRCA1 low H-score predicted worse OS in our Cox model adjusted for variables significantly affecting OS (adjusted HR = 1.58 95% CI [1.07-2.34], *p* = 0.02) while leading to worse DFS (HR = 1.44 95% CI [1.01-2.04], *p* = 0.04), though not in a Cox model adjusted for histology and stage (Table 2 and Figure 8, A and B left panel, respectively). No significant interaction between BRCA1 expression status and treatment arm was observed for DFS or OS prediction (data not shown).

**Figure 9:**
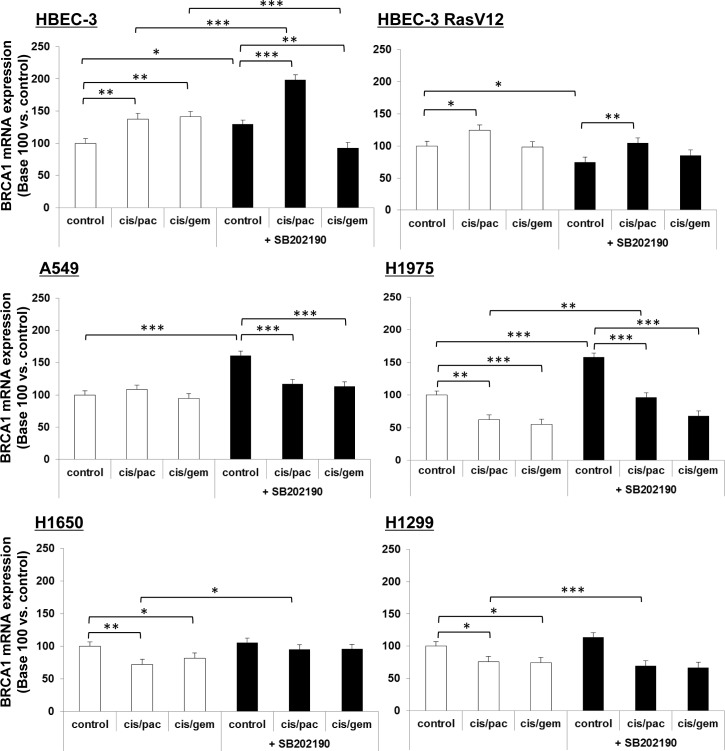
BRCA1 mRNA expression following p38 blocking or cisplatin/paclitaxel or cisplatin/gemcitabine treatment,in immortalized lung or lung cancer cell lines At 50% confluence, cell lines were incubated or not (control) for 1h with SB202190 (20μM) then with cisplatin (2μM) for 3h before incubation for 48h with paclitaxel (10nM) or gemcitabine (250nM). Following treatment, BRCA1 mRNA expression was assayed by qRT-PCR. Results were expressed in base 100, with 100 attributed to the control cells, *i.e.*, cells without any treatment for each cell line (one-way ANOVA, following post-hoc test of Fisher's LSD, **: *p* < 0.01, NS: non-significant).

In our experiments, the 8F7 clone did not enable reliable western blot (WB) analyses in lung cancer cell line extracts, despite claims that it functions for WB application. We then checked for any chemotherapy effects on cells in BRCA1 mRNA species, as measured by semi-quantitative qRT-PCR. We found only a slight increase in BRCA1 mRNA in non-tumorigenic cells following treatment by both doublets in HBEC-3 cells, potentiated by p38 inhibition, and only after paclitaxel-based doublet exposure in HBEC-3-RasV12 cells (Figure 9). In lung cancer cells, there was no variation (A549) or a slight decrease (H1975, H1650, H1299) of BRCA1 mRNA upon chemotherapy doublet treatment, with no potentiation by the p38 inhibitor (Figure 9).

Taking into account both the MSH2 and BRCA1 analyses, we subsequently tried to establish whether a combined expression signature of these two DNA repair proteins influenced the outcome of patients from the Bio-IFCT0002 trial. Among the 396 specimens, BRCA1 and MSH2 were simultaneously available in 204. Low MSH2 combined with high BRCA1 expression was more frequent in low-ECOG PS patients (*p* = 0.04) (Table 1). The proportion of low MSH2 cases was 50% (55 cases out of 110) in the low BRCA1 group, and 50% (55 cases out of 110) in the high BRCA1 group.

In both univariate and multivariate analysis, Cox models showed that high BRCA1 and low MSH2 expression appears to predict longer OS (HR = 0.51 [0.31-0.83], *p* = 0.006) and DFS (HR = 0.63 [0.42-0.95], *p* = 0.03), adjusted for clinical variables significantly influencing OS (histology, pathological stage, and gender) or DFS (histology and pathological stage), respectively (Table 2) (Figure 8, A and B right panel, respectively). A prognostic score was then constructed according to Cox models and validated by a bootstrap re-sampling strategy, which accurately defined three groups of early-lung cancer patients at low to high risk of recurrence and death despite perioperative cisplatin-based chemotherapy. These three patient groups were at “high risk” of death when BRCA1 was low and MSH2 high (*n* = 55, median OS >96 months, HR = 2.5, 95% CI [1.45-4.33], *p* = 0.001), at “low risk” when BRCA1 was high and MSH2 low (*n* = 67, median OS = 36.8 months ND, HR = 0.512.5, 95% CI [0.31-0.83], *p* = 0.006), and at “intermediate risk” in other combinations (*n* = 82, median OS = 73.4 *p* = 0.0596) (Figure 8C).

Finally, no significant interaction between low BRCA1 and high MSH2 expression status and treatment arm was observed in terms of DFS or OS prediction, again revealing this signature to be prognostic yet not predictive.

## DISCUSSION

We hereby report that the expression of two crucial DNA repair proteins, MSH2 and BRCA1, in early-stage NSCLC samples from patients who received preoperative platinum-based doublets in a Phase 3 trial, defines three distinct groups of high, intermediate, and low risk of death, according to their respective tumor expression of MSH2 and BRCA1.

Though the role of MSH2 is well established in several cancers, especially hereditary nonpolyposis colon cancer, with tumor immunohistochemistry used as a reliable tool for HNPCC its involvement in NSCLC is not yet clearly defined, with no report of any MSH2 gene mutations in such cancer subtype [35, 36]. Kamal *et al*. studied MSH2 protein expression using IHC in 673 tumor tissue paraffin-embedded samples from patients with resected NSCLC who received adjuvant platinum-based chemotherapy, reporting that MSH2 protein expression was a predictive factor [16]. In their work, high MSH2 expression was less frequent than in ours (38% *vs*. 53.1%, respectively), although different methods were used (high expression defined as a staining intensity >2, while we used a composite score). Patients with high MSH2 expression had an OS of 42 months (*vs*. 58 months for the others). In the current bio-IFCT-0002 study, patients with high MSH2 expression have an OS of 60 months (*vs*. over 100 months for the others). Several differences between these two studies must be emphasized: *i)* the sample size (356 *vs*. 673 for Kamal’s study), *ii)* the patients’ treatments (carboplatin/paclitaxel or cisplatin/gemcitabine combination in the IFCT-0002 trial, cisplatin-based adjuvant chemotherapy with a large percentage of older second-generation drugs in the IALT trial), *iii)* the chemotherapy administration schedule (patients in the IFCT-0002 trial received neo-adjuvant chemotherapy and tissue block samples were extracted during surgery, while IALT trial patients only received adjuvant chemotherapy). Nevertheless, our findings are in line with those of Kamal’s study: high MSH2 expression is a very strong prognostic factor.

We also report that using an additional marker may provide a better prognostic evaluation. Several DNA repair proteins could be used as prognostic or predictive biomarkers of response to chemotherapy or radiation in lung cancer patients. Kamal *et al*., for example, reported that combining both MSH2 and ERCC1 markers achieved better prediction of long-term chemotherapy benefit than using either one alone [16]. However, the ERCC1 monoclonal 8F1 antibody was disqualified in a more recent report from the same team due to specificity issues [37].

In the Bio-IFCT0002 study, high MSH2 expression combined with low BRCA1 expression indicated a risk of recurrence and death in early-stage lung cancer patients treated with perioperative chemotherapy. BRCA1 gene expression could be affected by allelic loss, gene mutations altering mRNA stability or promoter hypermethylation. We were not able to directly assess the influence of chemotherapy on tumor BRCA1 expression, but the lack of BRCA1 mRNA increase in bronchial cells or lung cancer cell lines on chemotherapy exposure indicates that neo-adjuvant chemotherapy had no influence on BRCA1 content in our patients, and that such treatment cannot account for the prognostic discrepancies with previously published papers. Indeed, in contrast to our findings, low BRCA1 expression was previously reported to be a potential biomarker for predicting the benefit of perioperative cisplatin-based chemotherapy [38]. Furthermore, high BRCA1 mRNA levels correlated with shorter OS in another study on chemo-naive patients with resected NSCLC [13]. All these findings were generated by means of mRNA analyses differing from ours using IHC analysis of protein expression. Other striking differences between our study and the literature are the proportion of squamous-cell carcinomas (SCC). SCC accounted for 36.5% of patients in the Bio-IFCT0002 trial versus 47% in the Taron study and 74% in the Rosell study [13, 38]. Moreover, Rosell reported a significantly higher content of BRCA1 mRNA in SCCs than in adenocarcinomas. Both of these studies also included Stage III patients (17.5% and 81%, respectively), rendering any comparison with the Bio-IFCT 0002 dataset challenging. The largest dataset of BRCA1 and MSH2 IHC (769 samples) was derived from the IALT adjuvant study [39]. which used tissue microarrays (TMAs) and digital-based automated scoring. In this series, comprising 57% SCC patients, BRCA1 expression was below the median expression score value in 65% of patients, *vs*. 41% in our series, suggesting significant differences in IHC scoring between the studies. It could, alternatively, indicate discrepancies in the influence of SCC proportion (57%), since the IALT study also reported higher BRCA1 expression in SCC samples. In their large patient sample, neither MSH2 nor BRCA1 were found to significantly influence OS, either in SCC or in the whole group, despite MSH2 expression being found to significantly predict DFS in SCC patients, though not in the whole population. These results contrast with those of earlier studies by the Rosell group (analyzing BRCA1 mRNA expression), thus suggesting the importance of epigenetic BRCA1 expression. The authors also contradict their own previously-published data (analyzing MSH2 IHC), suggesting the influence of TMA sampling biases, as compared with whole tumor section analysis [16] or suggesting some versatility of DNA-repair markers.

Whether combining MSH2 and BRCA1 markers could override conflicting data from the literature still requires confirmation in a prospective independent homogenous series of early-stage patients, as such discrepancies often result from differences in methodology and populations. However, this combination could create a more powerful prognostic model, as shown by the re-sampling internal validation of our MSH2/BRCA1 dual signature.

Our data thus demonstrate that specific biomarkers for DNA repair pathways can provide important prognostic information. Whether such information can guide oncologists in therapeutic decisions requires further prospective validation and the MSH2/BRCA1 signature deserves validation in patients included in the future neo-adjuvant clinical trials. The pharmacological modulation of DNA repair proteins has been suggested to increase the efficacy of DNA-interacting chemotherapy and radiotherapy, favoring tumor cell death primed by single- or double-strand DNA breaks [43]. Our data also provide a rationale to assess whether Poly(ADP-ribose) polymerase (PARP) inhibitors would increase chemotherapy efficacy in lung cancer patients with abnormal MSH2 or BRCA1 content, by a synthetic lethality mechanism.

## MATERIALS AND METHODS

### Patients and the Bio-IFCT 0002 trial

Between 2001 and 2005, the IFCT 0002 Phase 3 trial accrued 528 patients [5], comparing two platinum-based perioperative chemotherapy regimens, gemcitabine plus cisplatin or paclitaxel plus carboplatin, in Stage I or II NSCLC patients. Specific informed consent was obtained for biological studies (Bio-IFCT 0002) and approved by the trial’s appointed ethics committee (CPPRB of the Besançon University Hospital, France). Diagnostic biopsy samples before surgery were not available for comparison with post-operative samples used in this study since obtained by bronchial endoscopy and CT-guided trans-thoracic fine-needle aspiration biopsy, thus small-sized and used for routine histological characterization by standard immunohistochemistry (TTF1, p40, CK7, CK20 immunohistochemistry and HES, PAS-diastase colorations).

### Immunohistochemistry (IHC) and scoring

Paraffin-embedded surgical blocks were collected from 491 patients with incomplete histological response following neo-adjuvant chemotherapy. In this series, 7.2% of patients had complete histological response and only 461/528 (87%) specimens were available for IHC analysis [25]. Tumor paraffin-embedded blocks were processed as previously described [25]. Slides were incubated with primary antibody against BRCA1 (AbCam, clone 8F7, 1/400 - 20 min RT), MSH2 (Calbiochem, clone FE11, 1/200 - 60 min RT), or XRCC5 (Genetex, clone S10B1, 1/900 - 30 min RT), then revealed using the Novolink (Menarini, for BRCA1, MSH2) or Envision (Dako, for XRCC5) kits. Positive internal controls were systematically evaluated (normal epithelial cells). All slides were examined by one of four expert thoracic pathologists depending on the protein under analysis, one molecular biologist, and one clinician, all blinded to individual patient data. The staining intensity of each tumor-cell cluster, scored as 0 (negative), 1 (weak), 2 (moderate), or 3 (strong), at 40x magnification, was established by consensus among the three analysts (representative examples shown in Figure 1). The same overall IHC score was calculated for each staining by taking the sum of the staining intensity (0-3) multiplied by the distribution (0-100%), giving an H score between 0 and 300.

### O6MGMT promoter hypermethylation assay

Snap-frozen specimens were collected in centers possessing the facilities to bank frozen tissues at -80°C. The DNA yield from 208 samples was sufficient for independent polymerase chain reaction (PCR) amplifications for multiple molecular analyses, as previously published [26]. O^6^MGMT hypermethylation promoter was assayed by methylation-specific PCR (MS-PCR), with genomic DNA bisulfite modification performed using the Epitect™ kit (Qiagen, France), and PCR amplification as reported by Esteller *et al.* [41] PCR products were loaded onto 5% agarose gels, stained with Gel-Red™ (Interchim, France) and visualized under UV light.

### Cell culture and treatments

Isogenic immortalized human bronchial epithelial cells (HBEC) HBEC-3 and HBEC-3-RasG12V bronchial cells (42] were grown in KFSM (keratinocyte serum-free medium) supplemented with EGFr (0.2ng/ml) and BPE (bovine pituitary extracts, 25μg/ml) at 37°C in 5% CO2. The tumorigenic epithelial lung cancer-derived cells H1299, H1650, H1975, and A549 were from the American Type Culture Collection and used in early passages after reception. At 50% confluence, the cells were incubated or not for 1h with a p38 inhibitor, SB202190 (20μM), then cisplatin (2μM) was added for 3h before 48h of incubation with gemcitabine (250nM) or paclitaxel (10nM).

### DNAfragmentation

DNA fragmentation was assayed following the manufacturer’s procedures (Cell Death Detection ELISA plus kit™; Roche), with the cytoplasmic cell fraction used in the enzyme-linked immunosorbent assay, and 405nm optical density (OD, absorbance) was determined using a micro-plate reader following 5-min incubation with peroxidase substrate.

### Reverse transcription-quantitative real-time-PCR (RT-PCR)

Following extraction, RT-PCR was performed with each primer set (Table 3), as previously described [25]. RT-PCR data was normalized to the human S16. Relative quantification was calculated using the delta-deltaCt method.

**Table 3:** Sequence of Primers Used for PCR

Gene name	Primer sequence (5′-3′)
BRCA1	Forward: ACAGCTGTGTGGTGCTTCTGTG Reverse: CATTGTCCTCTGTCCAGGCATC
MSH2	Forward: GGAGGAGAGACTGCTGGAGA Reverse: TCCCTTTTTGCCTTTCAACA
XRCC5	Forward: GAAGGTGAAGATGGGTTGGA Reverse: AATTGGAGCCAATGGTCAGT
S16	Forward: CTGGAGCCAGTTCTGCTTCT Reverse: TCTGGTAATAGGCCACCAGG

### Immunoblotting

The antibodies used for IHC were the same as those used for WB. Whole-cell protein extracts were analyzed by WB, as previously described [25], with proteins detected with an enhanced chemiluminescence technique using the ECL kit™ (Promega).

### Immunofluorescence and image analysis

We performed immunofluorescence studies using the cytochrome C primary antibody from BD Biosciences diluted in 1% bovine serum albumin (BSA)/phosphate buffered saline (PBS) containing 0.3% triton X-100, added overnight at 1/50 and 4°C. The Alexa Fluor 555 (Invitrogen) secondary antibody was added for 1h at room temperature in 1% BSA/PBS. Coverslips were mounted with DAPI (Santa CruzTM) and image captured with high-throughput confocal microscopy (FluoView FV1000, Olympus™).

### Statistical analysis

The Bio-IFCT0002 study was a pre-planned ancillary and exploratory study. The characteristics of patients positive or not for each DNA repair protein on IHC, or exhibiting molecular alterations, were compared using chi-squared tests or Fisher’s exact tests for qualitative variables, and Student’s *t*-tests for quantitative variables. Associations between DNA repair protein expression and clinical characteristics were evaluated using chi-squared tests, Fisher’s exact tests, or Student’s *t*-tests.

Prognosis values for disease-free survival (DFS) and overall survival (OS), based on IHC scores, were assessed using Cox models. Interaction tests were used to evaluate predictive values. IHC scores were first studied as continuous variables ranging from 0 to 300. Median follow-up was estimated using the reverse Kaplan-Meier method. Multivariate Cox models were used to adjust for patients’ characteristics associated with the corresponding outcomes (DFS or OS) at *p* <0.20 in univariate analysis. In all models, the IHC scores were dichotomized (negative/positive) as indicated by a fractional polynomial analysis, the median value being selected in both MSH1 and BRCA1 analyses by this methodology. A two-step bootstrap re-sampling analysis was performed to validate the prognostic model, testing its stability and reproducibility [43] .The data was analyzed with SPSS software SPSS for Windows Version 15.0, Chicago, IL: SPSS, Inc., 2006), the mfp package of R software (mfp: multivariable fractional polynomials, R package Version 1.4.0, original by Gareth Ambler and modified by Axel Benner, 2007) and SAS software, Version 9.3 (SAS Institute Inc., Cary, NC, USA). The *in vitro* data are presented as mean ± standard error of the mean (SEM) of the three independent experiments. Statistical differences were assessed by Student’s paired *t*-test for single comparisons or one-way analysis of variance (ANOVA), followed by Dunnett’s multiple comparison test, to compare each condition from a given experiment with a single control (siNeg) (GraphPad Software, Inc. USA). Statistical significance was set at *p* ≤0.05.
